# Long-term bone metabolism outcomes in critically Ill patients with sepsis: a prospective series study

**DOI:** 10.1186/s12879-026-13318-2

**Published:** 2026-04-17

**Authors:** Qinyue Guo, Jiamei Li, Ruohan Li, Jing Xu, Kang Huo, Ying Pan, Gang Wang

**Affiliations:** 1 Department of Critical Care Medicine, The Second Affiliated Hospital of Xi’an Jiaotong University, Xi’an, Shaanxi 710004 China; 2 Department of Emergency Medicine, The First Affiliated Hospital of Xi’an Jiaotong University, Xi’an, Shaanxi 710061 China; 3 Department of Neurology, The First Affiliated Hospital of Xi’an Jiaotong University, Xi’an, Shaanxi 710061 China; 4 Department of Rheumatology and Immunology, The First Affiliated Hospital of Xi’an Jiaotong University, Xi’an, Shaanxi 710061 China; 5 Key Laboratory of Surgical Critical Care and Life Support, Xi’an Jiaotong University, Ministry of Education, Xi’an, China

**Keywords:** Vitamin D, Sepsis, Osteoporosis, Intensive care unit, Prospective

## Abstract

**Background:**

Although vitamin D deficiency has been linked to adverse outcomes in critically ill patients, its impact on sepsis-related short- and long-term complications, particularly skeletal sequelae, remains poorly understood. This study aimed to investigate the long-term bone metabolism outcomes in patients with sepsis and its association between vitamin D status.

**Methods:**

A retrospective baseline data collection and two prospective cohort analyses (with 5-year follow-ups) were conducted using data from 400 adults (18–70 years) admitted to the ICU (2008–2018) at the First Affiliated Hospital of Xi’an Jiaotong University in China. We first compared septic patients and non-septic patients, observing the relationship between sepsis and vitamin D levels. Next, we identified the long-term outcomes (osteoporosis) of patients after sepsis. Additionally, patients with sepsis were stratified into two cohorts based on baseline vitamin D levels. The outcomes in this cohort included all-cause 60-day mortality, and bone loss (osteoporosis). We conducted a statistical analysis using a 1:1 propensity score matching (PSM) to adjust for potential confounding variables.

**Results:**

A total of 400 patients were included in the study, of whom 214 were diagnosed with sepsis. Patients with sepsis were more likely to have lower serum 25(OH)D levels than non-sepsis subjects (12.6 ± 7.5 vs. 15.5 ± 7.7 ng/mL, *P* = 0.01). In assessing long-term outcomes, sepsis patients reflected a higher risk of developing osteoporosis than non-sepsis patients (62.2% vs. 16.6%, *P* = 0.01). Lower vitamin D levels after sepsis were also associated with a higher incidence of osteoporosis (*P* < 0.01).

**Conclusions:**

Vitamin D deficiency was a common finding during sepsis. In terms of long-term outcomes, the incidence of osteoporosis within 5 years of hospitalization was higher in sepsis patients, especially in patients with lower Vitamin D levels.

**Supplementary Information:**

The online version contains supplementary material available at 10.1186/s12879-026-13318-2.

## Background

With improved short-term survival, sepsis survivors now face significant long-term morbidity, including elevated risks of disability, chronic health deterioration, and post-discharge mortality, posing a major public health burden [1–8]. Among these sequelae, bone metabolism is increasingly recognized as a vulnerable target. The acute systemic inflammatory response in sepsis can mediate rapid bone loss, yet evidence on the trajectory of bone mass reduction during long-term recovery and its functional impact remains scarce [9].

Vitamin D is crucial for bone homeostasis, and its deficiency is common in critical illness. While low serum 25(OH)D levels have been associated with increased mortality in general ICU populations [10–13], interventional trials like VITdAL-ICU found no mortality benefit from correcting the deficiency in mixed critically ill patients [14]. This highlights the need to evaluate the role of vitamin D in specific patient subgroups, such as those with sepsis, and to look beyond mortality towards other pertinent long-term outcomes.

This study aimed to investigate the long-term bone metabolism outcomes in sepsis survivors and their association with vitamin D status. We hypothesized that sepsis survivors have a higher risk of developing osteoporosis and that vitamin D deficiency is associated with this risk. To test this, we first prospectively examined the association between sepsis and incident osteoporosis. Subsequently, within the sepsis cohort, we stratified patients by vitamin D status to analyze its impact on long-term skeletal outcomes.

## Methods

### Patients and study design

**Fig. 1:**
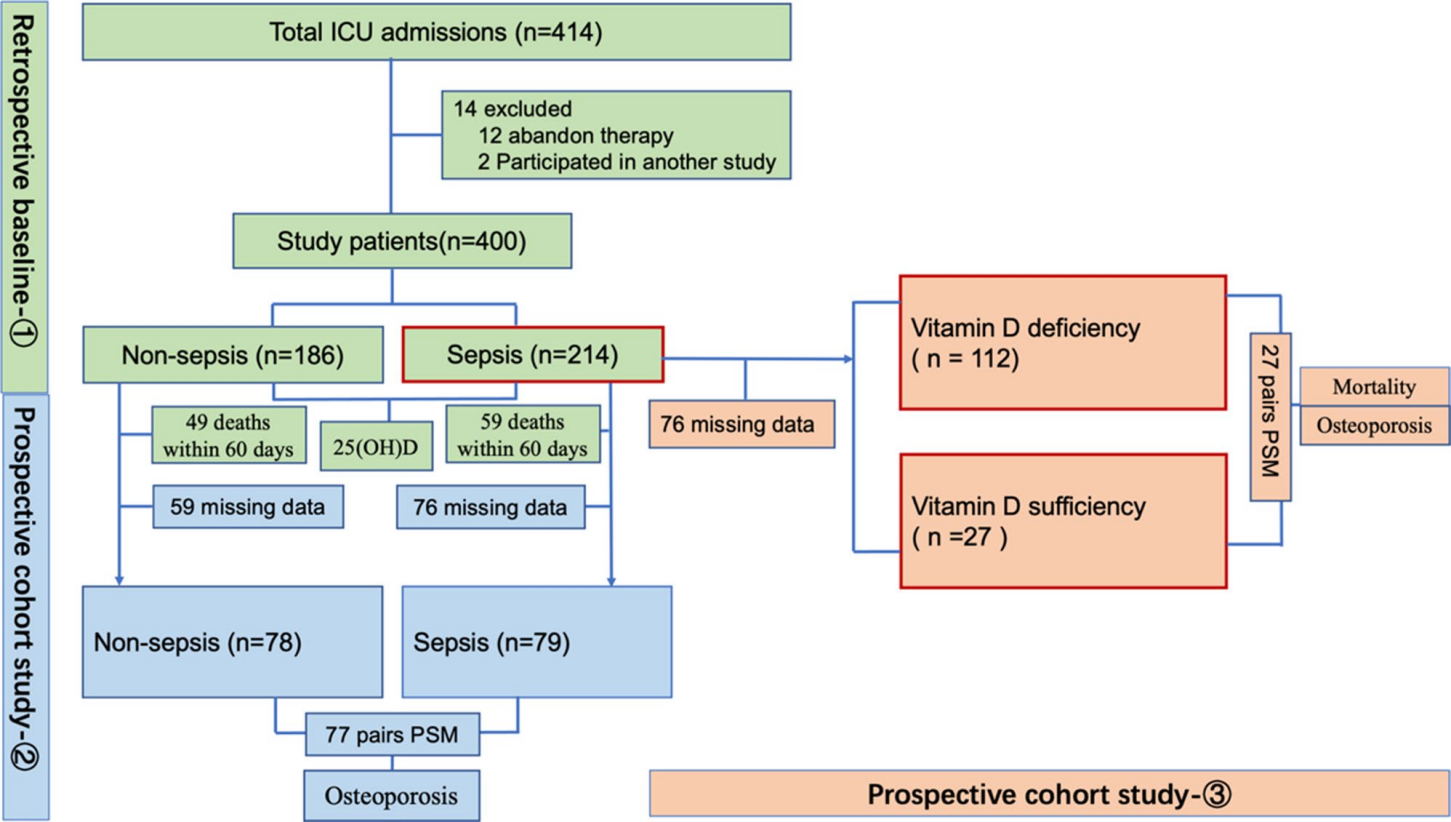
Flow diagram of the study

We conducted a retrospective baseline data collection and two prospective cohort studies at the First Affiliated Hospital of Xi’an Jiaotong University. Eligible participants were adults aged 18–70 years who were admitted to the ICU between January 2008 and November 2018. Our exclusion criteria were (1) pre-existing osteoporosis or metabolic bone disorders; (2) long-term glucocorticoid use (> 3 months); (3) pregnancy; and (4) incomplete baseline laboratory data. Participants received systematic follow-up every 12 months after being diagnosed with sepsis or non-sepsis upon admission to the ICU, with follow-up ending in December of 2023. Follow-up was conducted through structured face-to-face interviews, telephone follow-ups, and automated monitoring via electronic health records. We have developed a questionnaire specifically for this study and have uploaded it as a supplementary file. The outcomes were the first occurrence of osteoporosis or all-cause mortality. Patients were considered lost to follow-up if they failed to respond to three consecutive follow-up attempts, voluntarily withdrew from the study, or died during the follow-up period. Reasons for loss to follow-up were categorized as death, refusal to continue participation (explicit withdrawal by the patient or family), inability to contact the patient (e.g., due to invalid contact information or relocation without updated details), or other reasons such as interregional transfer or severe cognitive impairment. This study was approved by the Institutional Review Board of the First Affiliated Hospital of Xi’an Jiaotong University (Approval No. XJTU1AF2007LSK-014) and complied with the Declaration of Helsinki. The requirement for written informed consent was waived by the Institutional Review Board. This waiver was granted as the research involved no more than minimal risk, obtaining consent from the retrospective cohort was impracticable, and all data were handled using a de-identified coding system to protect participant confidentiality. To further clarify our data protection measures: All direct identifiers were removed from the analytical dataset and replaced with unique study codes, ensuring that statistical analysts could not identify individual participants. The master list linking codes to identities is securely stored and accessible only to the principal investigator. This study comprised a retrospective baseline data collection phase followed by two prospective longitudinal cohort analyses. Phase 1 (Retrospective baseline comparison: sepsis vs. non-sepsis vitamin D levels), Phase 2 (Prospective Cohort 1: sepsis vs. non-sepsis osteoporosis risk), and Phase 3 (Prospective Cohort 2: Vitamin D-stratified outcomes within sepsis patients). The outcomes in this cohort included all-cause day-60 mortality rates, and osteoporosis. Figure 1 provides a detailed schematic of the study design, including enrollment criteria and outcome measures.

### Data collection

We collected data from the medical record information system as follows: (1) demographics (sex, age, and body mass index) and living habits (smoking and alcohol misuse); (2) comorbidities (cardiovascular diseases that included coronary heart disease, hypertension, and valvular heart disease; chronic liver diseases that included chronic hepatitis and cirrhosis; and chronic respiratory disease referred to as chronic obstructive pulmonary disease, bronchitis, and interstitial lung disease); (3) scores were designated as an acute physiology and chronic health evaluation II (APACHE II) score and a sequential organ failure assessment (SOFA) score; and (4) laboratory findings comprised white blood cell count (WBC), lymphocyte count, neutrophils, C-reactive protein (CRP), procalcitonin (PCT), platelet count, parathyroid hormone, serum calcium, and serum phosphorus.

Blood samples were collected at hospital admission and weekly during hospitalization, and serum was separated by centrifugation (2000 ×*g* at 4 °C for 10–15 min) within 30 min of collection, stored at 2–8 °C, and analyzed for 25(OH)D concentrations within 4 h using a Beckman Coulter UniCel DxI 800 system following the manufacturer’s protocols (intra-assay CV, < 5%). The laboratory reports of serum calcium and phosphorus and the outcomes were collected through an annual review of health records and questionnaires each year. Telephone verification was performed for event-free cases at study closure, and to minimize attrition, we implemented bimonthly reminder calls during the period of follow-up.

### Definition

For sepsis patients, baseline serum 25(OH)D concentrations were defined as the time of initial sepsis diagnosis, whereas for non-sepsis patients, the value measured within 24 h of hospital admission. We defined sepsis as life-threatening organ dysfunction created by a dysregulated host response to infection, organ dysfunction was identified as an acute change in total SOFA score ≥ 2 points consequent to the infection (sepsis 3.0) [15], and vitamin D deficiency was characterized as serum 25(OH)D concentrations below 20 ng/mL with the Endocrine Society guidelines [16]. For patients with multiple follow-up measurements of serum calcium, phosphorus, and 25(OH)D levels, we applied their final laboratory values for analysis (values measured at the last follow-up visit).

Osteoporosis was diagnosed based on bone mineral density (BMD) measurement using dual-energy X-ray absorptiometry (DXA) using the device of Hologic Discovery Wi (Hologic, Inc., Bedford, USA). BMD was assessed at the lumbar spine (L1-L4) and/or femoral neck. A T-score ≤ -2.5 standard deviations was defined as osteoporosis, according to WHO criteria [17, 18]. DXA scans were performed at the hospital’s metabolic bone center following standardized quality control procedures. Follow-up DXA was scheduled at 12–24-month intervals or when clinically indicated (e.g., low-trauma fracture, significant height loss). The time to an osteoporosis event was calculated from baseline to the date of the first event. Patients without a documented diagnosis of osteoporosis or osteoporotic events before death were excluded from our analysis and considered lost to follow-up.

### Statistical analysis

We conducted statistical analyses using SPSS Statistics Version 29 (IBM, Armonk, NY, USA). Continuous variables are presented as the mean ± SD, with the follow-up duration presented as the median (interquartile range, IQR). A formal sample size calculation was performed a priori. Since direct estimates of post‑sepsis osteoporosis incidence were lacking in the literature, our assumptions were derived from indirect evidence and clinical reasoning. We posited that the profound inflammatory burden of sepsis would confer a substantially higher risk of bone loss compared with non‑septic critical illness. This assumption was indirectly supported by an article [19] reporting a 40% osteoporosis incidence in a COVID‑19 cohort. Based on these considerations, with α = 0.05 and β = 0.20 (power = 80%), the calculation indicated a minimum requirement of 80 patients per group. Our final recruited cohorts met or exceeded this target for all primary comparisons.

We made comparisons between study cohorts with Student’s *t* test or Mann-Whitney U test. Categorical variables are presented as counts (percentages), and differences between them were estimated using the Chi-squared or Fisher exact test, as appropriate. To control for baseline confounding factors, a propensity score matching (PSM) approach was employed. The final set of matching variables—age, sex, smoking history, cardiovascular disease, APACHE II score, and SOFA score—was selected based on their established clinical relevance to both sepsis/vitamin D metabolism and bone outcomes, as supported by existing literature. To maximize matching quality within our cohort, we employed 1:1 nearest-neighbor matching with replacement to optimize matching quality given the sample size constraints, allowing some individuals with substantial baseline differences to be excluded from the matched analytical cohort, using a caliper of 0.1 times the standard deviation of the propensity score logit. Balance was assessed using Standardized Mean Differences (SMD), with SMD < 0.1 indicating adequate balance. All reported analyses are based on the matched sample, with the effective sample size stated in the results. All tests of significance were two-sided, with *p* values < 0.05 considered to be statistically significant.

## Results

### Relationship between sepsis and vitamin D

A total of 414 patients were admitted to the Department of Critical Care Medicine at the First Affiliated Hospital of Xi’an Jiaotong University between January 2008 and November 2018. After excluding 14 patients (12 due to treatment discontinuation and two enrolled in concurrent clinical trials), 400 patients were included. Table 1 summarizes the baseline clinical characteristics of the study population. In total, 214 patients were diagnosed with sepsis, and 186 served as non-sepsis controls. Sepsis patients were significantly older than non-sepsis patients (mean age 60.7 ± 16.8 years vs. 56.2 ± 17.1 years, *P* < 0.01). No significant differences were observed between the two groups in terms of sex distribution, body mass index (BMI), or lifestyle habits (e.g., smoking and alcohol consumption). Additionally, comorbidities, baseline laboratory parameters, and disease severity scores (e.g., APACHE II and SOFA) did not demonstrate significant differences between patients with sepsis and non-sepsis patients.

**Table 1:** Baseline characteristics of ICU patients with sepsis and non-sepsis

	Sepsis ( *n* = 214)	Non-sepsis ( *n* = 186 )	*P* value	*P* value Matched^a^(186)
Age, yr, mean ± SD	60.7 ± 16.8	56.2 ± 17.1	0.01	0.06
Male sex	68 (31.7%)	73 (39.2%)	0.03	0.08
BMI, kg/m^2^	22.4 ± 4.8	22.5 ± 3.5	0.80	0.08
Smoking	71 (33.1%)	37 (19.9%)	0.07	0.07
Alcohol misuse	45 (21.0%)	26 (13.9%)	0.18	0.22
**Comorbidities**				
Chronic liver disease	43 (20.0%)	27 (14.5%)	0.78	0.53
Chronic respiratory disease	16 (7.5%)	6 (3.2%)	0.26	0.18
Cardiovascular disease	45 (21.0%)	48 (25.8%)	0.07	0.08
Diabetes	25 (11.7%)	17 (9.1%)	0.25	0.29
Chronic renal disease	31 (14.5%)	26 (13.9%)	0.22	0.45
**Scores**				
APACHE II score	22.7± 8.2	21.1± 6.9	0.07	0.07
SOFA score	9.3 ± 4.4	8.5 ± 4.1	0.10	0.13
**Laboratory results**				
Serum phosphate (mmol/L)	1.3 ± 0.2	1.3 ± 0.1	0.81	0.82
Serum calcium (mmol/L)	2.1 ± 0.1	2.0 ± 0.2	0.55	0.50
25(OH)D (ng/mL)	12.6 ± 7.5	15.5± 7.7	**0.04**	**0.01**

Definition of abbreviations: BMI, Body Mass Index; WBC, white blood cell count; APACHE II, Acute Physiology and Chronic Health Evaluation II; SOFA, Sequential Organ Failure Assessment Score

Data are presented as the mean ± SD or number (percentage) of patients

^a^Of 401 patients, 186 pairs were matched

To address potential confounding factors, we performed propensity score matching (PSM) using a 1:1 ratio. After matching, 186 sepsis patients were paired with 186 non-sepsis controls. The matched cohorts exhibited similar baseline characteristics, with no statistically significant differences in age (*P* = 0.06) or other demographic/clinical variables (all *P* > 0.05). Sepsis patients were more likely to have lower serum 25(OH)D levels than non-sepsis patients, for either the unadjusted (*P <* 0.05) or PSM results (*P* = 0.01). The average serum 25(OH)D levels in the sepsis versus non-sepsis patients were 12.6 ± 7.5 ng/mL and 15.5 ± 7.7 ng/mL, respectively, with both groups demonstrating states of vitamin D deficiency (Table 1).

### Causal relationship between sepsis and osteoporosis (prospective cohort study)

During the 5-year follow-up period, a total of 157 patients from our study completed longitudinal assessments for osteoporosis (Fig. 1). 79 patients had a previous history of sepsis (among whom 49 [62.2%] developed osteoporosis), and 78 patients were in the non-sepsis control group (among whom 13 [16.6%] developed osteoporosis). There was no difference in the duration of follow-up between the two groups (*P* > 0.05). The median follow-up duration was 30 months (IQR, 18–50) in the sepsis group and 35 months (IQR, 20–60) in the control group, reflecting right-skewed distributions consistent with the observed mean ± SD values (36.5 ± 18.8 vs. 40.3 ± 22.4 months, respectively). As shown in Supplementary Table 1, the baseline characteristics of the retained and lost-to-follow-up groups were similar, and differences were not significant (*P* > 0.05).

Table 2 depicts the clinical characteristics of patients in this prospective cohort study. The mean age of the sepsis patients was 61.9 years (SD 15.4 years), which was significantly higher than the mean age of the non-sepsis patients (56.3 ± 16.5 years) (*P* = 0.03). Other data, including sex, BMI, and living habits, did not differ between the two groups (all *P* > 0.05). APACHE II and SOFA scores revealed similar degrees of illness severity in the two groups (*P* > 0.05).

**Table 2:** Compared the long-term outcomes of patients who survived from sepsis and non-sepsis

	Sepsis ( *n* = 79)	Non-sepsis ( *n* = 78)	*P* value	*P* value Matched^a^(77)
Age, yr, mean ± SD	61.9 ± 15.4	56.3 ± 16.5	0.03	0.08
Male sex	22 ( 27.8%)	31 (39.4%)	0.18	0.23
BMI, kg/m^2^	22.9 ± 5.6	22.5 ± 3.5	0.77	0.78
Smoking	27( 34.2%)	19( 24.4%)	0.44	0.48
Alcohol misuse	18(22.8%)	12 (15.4%)	0.43	0.49
Follow-up duration, months	30 (18–50)	35 (20–60)	0.25	0.27
**Comorbidities**				
Chronic liver disease	15 (19.0%)	11 (14.1%)	0.83	0.99
Chronic respiratory disease	5 (6.3%)	4 (5.1%)	1.00	0.99
Cardiovascular disease	39(49.3%)	27 (34.6%)	0.40	0.50
Diabetes	14(17.7%)	6(7.7%)	0.90	0.92
Chronic renal disease	10(12.7%)	12(15.4%)	0.36	0.48
**Status of the patient**				
Corticosteroids	58(73.4%)	47(60.3%)	0.09	0.09
Neutropenia	14(17.7%)	14(17.9%)	0.53	0.53
Immunoglobulin deficiency	0(0%)	3(3.8%)	0.06	0.08
**Scores**				
APACHE II score	22.5 ± 5.2	21.3 ± 4.3	0.21	0.63
SOFA score	9.8 ± 3.4	8.9 ± 4.0	0.11	0.71
**Laboratory results(follow-up)**				
Serum phosphate (mmol/L)	1.4 ± 0.2	1.3 ± 0.1	0.78	0.80
Serum calcium (mmol/L)	2.2 ± 0.2	2.3 ± 0.2	0.66	0.68
25(OH)D (ng/mL)	18.6 ± 7.5	23.5± 7.7	**0.03**	**0.01**
**Outcome**				
Osteoporosis	49(62.2%)	13(16.6%)	**0.01**	**0.01**

Definition of abbreviations: BMI, Body Mass Index; APACHE II, Acute Physiology and Chronic Health Evaluation II; SOFA, Sequential Organ Failure Assessment Score.Data are presented as the mean ± SD or number (percentage) of patients.The follow-up duration presented as the median (IQR). Laboratory results(follow-up)measured at the last follow-up visit

^a^Of 157 patients, 77 pairs were matched

To reduce the bias in the two groups, we performed a PSM analysis with 77 sepsis patients successfully matched with 77 non-sepsis patients. After being matched for propensity score, the difference in age between the two groups was no longer statistically significant (*P* > 0.05). When assessing long-term outcomes, patients with sepsis were more likely to develop osteoporosis, with the same trend shown for the unadjusted (*P* = 0.01) and PSM analyses (*P* = 0.01). Notably, during the follow-up period, we also found that sepsis patients were more likely to have low serum 25(OH)D levels (*P* < 0.05) in spite of serum calcium and phosphorus levels within the normal range (*P* > 0.05) (Table 2).

### Causal relationship between vitamin D deficiency and outcomes in patients with sepsis (prospective cohort study)

Of the 214 sepsis patients from the study, 76 individuals were lost to follow-up. Therefore, a total of 139 patients were analyzed in the prospective cohort study for the association between vitamin D deficiency and outcomes (Fig. 1), consisting of 112 patients (80.6%) with vitamin D deficiency and 27 patients with vitamin D sufficiency (19.4%). The vitamin D deficiency group exhibited a median follow-up of 35 months (IQR, 12–47), versus the vitamin D sufficiency group (32 months; IQR, 20–50), despite comparable mean values (31.5 ± 22.2 vs. 38.4 ± 18.8 months, *P* > 0.05). Characteristics of the patients were stratified according to 25(OH)D levels (as depicted in Table 3**)**. There was no difference between the two groups when analyzed for age, sex, BMI, or comorbidities (*P* > 0.05). For laboratory results, the patients in the vitamin D deficiency group were more likely to exhibit lower lymphocyte count levels (*P* = 0.03), higher C-reactive protein levels (*P* = 0.01), and higher procalcitonin levels (*P* = 0.04) than the patients in the vitamin D sufficiency group. Furthermore, sepsis patients with vitamin D deficiency had significantly higher SOFA scores than sepsis patients with vitamin D sufficiency (*P* = 0.01). In addition, patients with vitamin D deficiency had a higher incidence of osteoporosis (44.6% vs. 3.7%, *P* = 0.01). However, there was no difference in all-cause day-60 mortality rates between the two groups (40.2% vs. 51.9%, *P* = 0.54) (Table 3).

**Table 3:** Characteristics of septic patients according to vitamin D status

	Vitamin D deficiency ( *n* = 112) < 20 ng/mL	Vitamin D sufficiency ( *n* = 27) > 20 ng/mL	*P* value	*P* value Matched^a^(27)
Age, yr, mean ± SD	60.9 ± 16.8	57.4 ± 18.9	0.24	0.66
Male sex	67	19	0.74	0.78
BMI, kg/m^2^	23.0 ± 4.3	22.3 ± 4.1	0.48	0.56
Smoking	57	13	0.60	0.69
Alcohol misuse	39	7	0.42	0.87
Follow-up duration, months	35(12–47)	32(20–50)	0.14	0.67
**Comorbidities**				
Chronic liver disease	37	11	0.69	0.65
Chronic respiratory disease	9	2	1.00	0.99
Cardiovascular disease	88	20	0.52	0.67
Diabetes	29	13	0.09	0.46
Chronic renal disease	26	13	0.05	0.43
**Status of the patient**				
Pregnancy	1	2	0.11	0.45
Corticosteroids	142	35	0.70	0.78
Neutropenia	38	4	0.06	0.45
Immunoglobulin deficiency	3	1	0.87	0.89
**Scores**				
APACHE II score	23.6 ± 8.1	21.6 ± 9.4	0.15	0.34
SOFA score	9.8 ± 4.5	8.0 ± 3.9	**0.01**	0.32
**Laboratory results**				
WBC (x 10^9^/L)	11.6 ± 10.0	11.3 ± 12.4	0.89	0.88
Lymphocyte count (x 10^9^/L)	0.7 ± 0.6	0.9 ± 1.4	**0.03**	0.23
Neutrophil count (x 10^9^/L)	10.1 ± 8.3	9.3 ± 7.1	0.54	0.68
C-reactive protein (mg/L)	121.1 ± 88.1	85.6 ± 81.4	**0.01**	0.08
Procalcitonin (ng/ml)	10.8 ± 18.4	5.3± 16.4	**0.04**	0.54
Platelet (x 10^9^/L)	126.5 ± 90.1	135.7±108.9	0.59	0.57
Parathyroid hormone(pg/ml)	13.1 ± 7.6	14.8 ± 5.6	0.40	0.67
**Outcome**				
All-cause day-60 mortality	45	14	0.54	0.80
Osteoporosis	50 (44.6%)	1 (3.7%)	**0.01**	**0.01**

Definition of abbreviations: BMI, Body Mass Index; WBC, white blood cell count; APACHE II, Acute Physiology and Chronic Health Evaluation II; SOFA, Sequential Organ Failure Assessment Score. Data are presented as the mean ± SD or number (percentage) of patients. The follow-up duration presented as the median (IQR)

Laboratory data were obtained at sepsis diagnosis for the sepsis group and within 24 h of admission for the non-sepsis group, respectively

^a^Of 139 patients, 27 pairs were matched

**Fig. 2:**
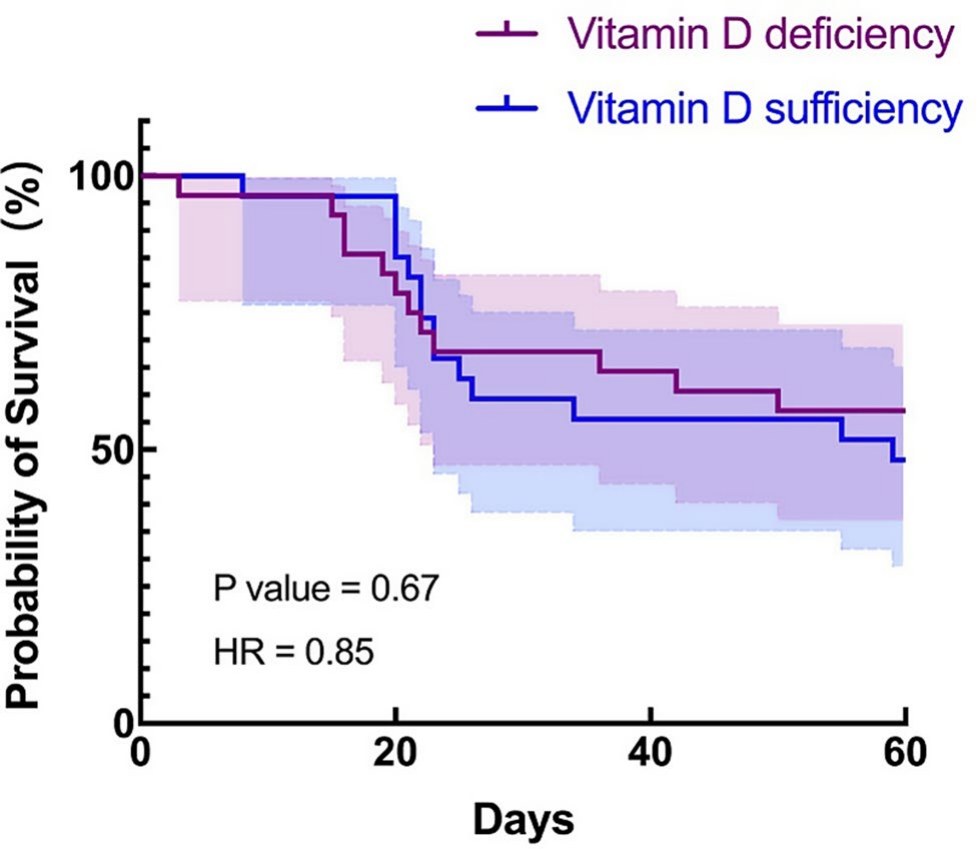
Kaplan-Meier survival curve of 27 pairs of septic patients stratified by the vitamin D status

To reduce the bias in the two groups, we performed a PSM analysis with 27 Vitamin D sufficiency septic patients with 27 Vitamin D deficiency septic patients. After being matched for propensity score, patients with vitamin D sufficiency were still found to be at a higher risk of developing osteoporosis (OR 20.8, 95% CI 2.8 to 229.9, *P* = 0.01) (Table 4), however, there was no significant difference of all-cause day-60 mortality (OR 0.64, 95% CI 0.2 to 1.8, *P* = 0.80) (Table 4 and Fig. 2).

**Table 4:** Analysis of septic patients according to vitamin D status associated with 60-day mortality rate and osteoporosis

	Crude OR (95% CI)	*P* value	Propensity-matched^a^ OR (95% CI)	*P* value
Outcome				
All-cause day-60 mortality	0.62 (0.3–1.5)	0.54	0.64 (0.2–1.8)	0.80
Osteoporosis	20.9(3.6–220.4)	**0.01**	20.8 (2.8–229.9)	**0.01**

Definition of abbreviations: CI, Confidence interval; OR, Odds ratio

^a^Of 139 patients, 27 pairs were matched

## Discussion

With this study, we demonstrated that patients with sepsis exhibited lower 25(OH)D levels and that they displayed a higher risk of developing osteoporosis. Additionally, sepsis with vitamin D deficiency may contribute to higher incidence of osteoporosis. Collectively, these findings suggest that sepsis may directly impair vitamin D metabolic pathways—a hypothesis that warrants further investigation into the underlying mechanisms involved. Emerging evidence from critical care research has established a bidirectional association between sepsis and vitamin D homeostasis. Meta-analyses consistently reveal that hypovitaminosis D correlates with adverse clinical trajectories in sepsis, including heightened susceptibility to infection, prolonged hospitalization, acute kidney injury, and elevated mortality risk [20–22]. While these studies primarily focused on one-time measurements of vitamin D, in our investigation, we uniquely examined the temporal dynamics of vitamin D metabolism following sepsis. In addition, we ascertained that vitamin D deficiency was not only prevalent during the acute phase of sepsis but also persisted over a 5-year follow-up period—with sepsis associated with sustained hypovitaminosis D compared with matched non-sepsis controls. Furthermore, extant studies have not entailed the late incidence (taking into account the occurrence) of osteoporosis in patients with a history of sepsis. In our study, we discerned that patients with sepsis reflected a higher risk of bone loss and osteoporosis. Osteoporosis significantly increases the risk of fractures, severely compromising patients’ quality of life [18]. Future studies with longer follow-up durations are warranted to further evaluate the association between sepsis and fracture risk.

The association between vitamin D deficiency and mortality in sepsis is still debated. Evidence shows that hypovitaminosis D correlates with increased 30-day mortality in patients with severe sepsis [23]. De Pascale further identified a level of 25(OH)D < 7 ng/mL as an independent mortality predictor [24]. However, in the observational FINNAKI cohort study [25], vitamin D deficiency (defined as 25(OH)D < 20ng/mL) at ICU admission was not associated with hospital mortality in patients with severe sepsis or septic shock. The observed discrepancies across studies may be attributable to heterogeneity in patient populations and inconsistent definitions of vitamin D deficiency. In our study, we applied classification criteria for vitamin D deficiency—i.e., 25(OH)D < 20 ng/mL—as recommended by the Endocrine Society clinical practice guidelines, and conducted a 5-year follow-up. Nevertheless, we found that the relationship between vitamin D deficiency and mortality remained insignificant. Future research with larger sample sizes and multiple centers may further clarify this relationship.

Although we did not observe a difference in mortality in sepsis patients with vitamin D deficiency, we did confirm through long-term follow-up that sepsis patients manifested a worse long-term quality of life as indicated by a higher incidence of osteoporosis. Given the paucity of research on post-sepsis osteoporosis, we expanded our initial investigation by analyses of longitudinal outcomes in this understudied population. We divided patients with sepsis into two groups based on their level of vitamin D and found that in the follow-up period, osteoporosis after sepsis was related to vitamin D deficiency; this suggests that vitamin D deficiency may constitute an important factor leading to osteoporosis after sepsis. This conclusion provided complementary data from the long-term outcomes in sepsis, while it also provided novel concepts as to whether to supplement vitamin D after sepsis. Our findings support a novel rationale for post-sepsis vitamin D supplementation—not merely to correct acute deficiency but to mitigate long-term skeletal morbidity through targeted interventions.

Our results further confirm the findings of multiple previous observational studies, indicating a close association between sepsis and vitamin D deficiency. There is currently much debate regarding the utility of vitamin D supplementation after critical illness. A study from the southeastern United States confirmed that a vitamin D–replete state may reduce costs and confer survival advantages in critically ill patients, and the authors recommended the routine measurement of 25(OH)D levels in ICU patients—with treatment of vitamin D deficiency when present [26]. However, the authors of the VITdAL-ICU Randomized Clinical Trial concluded that among critically ill patients with vitamin D deficiency and compared with placebo, administration of high-dose vitamin D_3_ did not reduce hospital length of stay, hospital mortality, or 6-month mortality [14], this finding consistent with our observation. Notably, our longitudinal data expanded this discourse by revealing an increased risk of osteoporosis in sepsis survivors compared with non-sepsis controls during a 5-year follow-up. Therefore, based on previous studies and our current findings, although the effect of vitamin D on mortality in critically ill patients remains inconclusive, its supplementation may provide clinical benefits for sepsis patients by reducing osteoporosis risk and improving long-term quality of life. Further validation through well-designed, randomized controlled trials is, however, warranted.

Regarding the specific molecular mechanisms linking sepsis and vitamin D deficiency, our follow-up data showed that vitamin D deficiency may be critical to the overall process by involving non-phosphorus metabolic pathways. We found that sepsis patients were more likely to have low serum 25(OH)D levels after discharge in spite of normal serum calcium and phosphorus levels. We plan to investigate these mechanisms in greater detail in our future investigations. Accumulating evidence underscores the pivotal role of vitamin D homeostasis in sepsis pathophysiology. A pathophysiologic cause underlying sepsis in vitamin D deficiency may be related to innate immune dysfunction [27]. Moreover, a reduction in 25(OH)D at the tissue level may lower its pleiotropic effects on immune regulation and mucosal and endothelial function [28]. In another study, investigators confirmed that higher 25(OH)D levels were associated with lower systemic levels of proinflammatory cytokines such as IL-1 and IL-6 [29]. In the current study, we also ascertained that vitamin D deficiency was often accompanied by higher biomarkers of infection in patients with sepsis, and the more severe the vitamin D deficiency, the more obvious the difference in infection indicators. Reciprocally, infection can also lead to vitamin D deficiency. A study of serum concentrations of 25(OH)D in patients with severe sepsis revealed a high prevalence of vitamin D deficiency in three-quarters of the patients who had severe sepsis [23], and our study also verified this finding. However, additional, in-depth research is required to clarify whether vitamin D deficiency causes sepsis or whether sepsis aggravates vitamin D deficiency, and whether these interactions occur through the same mechanism or different mechanisms.

The first limitation of this study was a high rate of loss to follow-up; however, a comparison of baseline characteristics between participants lost to follow-up and those retained in the study demonstrated no significant differences; this therefore partially mitigated potential selection bias. The second limitation was our inability to determine the sequence of data collection and the onset of sepsis when we collected the vitamin D levels, but we nevertheless could still speculate on the correlation between sepsis and vitamin D levels. The third limitation was that although our study identified both sepsis and vitamin D deficiency as significant risk factors for osteoporosis in septic patients, the short follow-up duration (5 years) limited our ability to establish associations with other bone-related disorders. Such incomplete aspects underscore the necessity for larger-scale cohorts and extended observation periods to adequately capture clinically meaningful differences in post-sepsis skeletal outcomes. In addition, in this study, patients’ sun exposure behaviors and use of vitamin D/calcium supplements during the follow-up period were not adjusted for as covariates, which may have some influence on the outcome estimates. Fourth, as a single-center study, our findings may be influenced by local patient demographics and clinical practices, which could limit their generalizability. Multi-center studies are needed for validation. The last limitation of our study is the exclusion of individuals over 70, which may restrict generalizability to the very old population, as this group often has multiple comorbidities that could confound the relationship between sepsis and bone mineral density.

## Conclusion

In conclusion, we herein verified vitamin D deficiency as a common finding in patients with sepsis. The incidence of osteoporosis was higher after sepsis, especially in patients with lower Vitamin D levels. Vitamin D deficiency may therefore play a role in this process through non-phosphorus metabolism pathways.

## Supplementary Information

Below is the link to the electronic supplementary material.

Supplementary Material 1

Supplementary Material 2

Supplementary Material 3

## Data Availability

No datasets were generated or analysed during the current study.
