# Supplementary material for: Long-term bone metabolism outcomes in critically Ill patients with sepsis: a prospective series study
Source: BMC Infect Dis. 2026 Apr 17;26:1045. doi: 10.1186/s12879-026-13318-2 (PMC13217769; doi:10.1186/s12879-026-13318-2)
Supplement: Supplementary file 1 — Supplementary Material 1 [file 12879_2026_13318_MOESM1_ESM.docx]

Long-Term Follow-Up Questionnaire for Sepsis Patients: Bone Health and Metabolism

Questionnaire ID:________

Follow-up Time Point:□ 1 Year □ 2 Years □ 3 Years □ 4 Years □ 5 Years

Date of Interview: ____/____/________

Method of Interview: □ Outpatient Visit □ Telephone Interview □ Electronic Health Record (EHR) Review

Section A: Basic Information

| Item | Details |

| Patient Name |

| Gender| □ Male □ Female |

| Age (at follow-up)| ____ years |

| Contact Phone Number |

| Hospital Admission ID |

| ICU Admission Date | ____/____/________ |

| Hospital Discharge Date | ____/____/________ |

| Diagnosis Group| □ Sepsis □ Non-Sepsis (Control) |

Section B: Lifestyle and Habits

1. Smoking History:

□ Never Smoked

□ Former Smoker (Quit Date: ____/____/________)

□ Current Smoker (Average ____ cigarettes/day)

2. Alcohol Consumption:

□ Never Drinks

□ Occasional Drinker (<1 time/week)

□ Frequent Drinker (≥1 time/week)

3. Daily Sun Exposure:

□ <30 minutes/day

□ 30–60 minutes/day

□ >60 minutes/day

4. Exercise Habits:

□ Sedentary (Little to no exercise)

□ Light Exercise (e.g., walking)

□ Moderate Exercise (e.g., brisk walking, cycling)

□ Vigorous Exercise (e.g., running, swimming)

5. Use of Calcium or Vitamin D Supplements:

□ Yes (Type: ________, Dose: ________, Frequency: ________)

□ No

Section C: Health Status and Comorbidities

1. Has a doctor ever diagnosed you with osteoporosis or low bone density (osteopenia)?

□ Yes (Date of diagnosis: ____/____/________)

□ No

□ Unsure

2. History of Fractures (after hospital discharge):

□ Yes (Location: ________, Date: ________, Cause: ________)

□ No

3. Long-term use of corticosteroid medications (e.g., Prednisone):

□ Yes (Duration: ____ months, Dose: ________)

□ No

4. Other Comorbidities (Check all that apply):

□ Hypertension

□ Diabetes Mellitus

□ Coronary Heart Disease

□ Chronic Kidney Disease

□ Chronic Liver Disease

□ Chronic Respiratory Disease (e.g., COPD)

□ Other: ________

Section D: Bone Health and Symptoms

1. Have you experienced any bone pain or joint pain in the past year?

□ Yes (Location: ________)

□ No

2. Have you noticed a loss of height or development of a stooped posture (kyphosis) in the past year?

□ Yes

□ No

3. Have you undergone a Bone Mineral Density (BMD) test (DXA scan) since discharge?

□ Yes (Most recent test date: ____/____/________, T-Score: ________)

□ No

Section E: Laboratory Results (Please attach most recent report)

| Test | Value | Unit | Date of Test |

| Serum 25-Hydroxyvitamin D [25(OH)D]| ng/mL | ____/____/________ |

| Serum Calcium (Corrected) | mmol/L | ____/____/________ |

| Serum Phosphate| mmol/L | ____/____/________ |

| Parathyroid Hormone (PTH)| | pg/mL | ____/____/________ |

| Alkaline Phosphatase (ALP) | U/L | ____/____/________ |

| C-Reactive Protein (CRP) | mg/L | ____/____/________ |

| White Blood Cell Count (WBC) | ×10⁹/L | ____/____/________ |

Section F: Adverse Events and Outcomes

New Fracture Event: □ Yes (Date/Location/Cause: ______________) □ No

- Re-hospitalization: □ Yes (Primary Reason: ________________) □ No

- Mortality: □ Yes (Date of Death: ______, Presumed Cause: _____) □ No

Section G: Additional Notes

(For any other relevant information or patient comments)

Investigator's Signature:________

Patient/Next-of-Kin Confirmation:_______

Date:___/____/________

Instructions for Use:

- This questionnaire should be administered at each annual follow-up point.
- For data obtained via EHR review, please note the source and date of extraction.
- The "Laboratory Results" section can be completed by reviewing the patient's most recent medical records.
- Please ensure all applicable boxes are checked and fields are completed as thoroughly as possible.
