# Supplementary material for: Long-term bone metabolism outcomes in critically Ill patients with sepsis: a prospective series study
Source: BMC Infect Dis. 2026 Apr 17;26:1045. doi: 10.1186/s12879-026-13318-2 (PMC13217769; doi:10.1186/s12879-026-13318-2)
Supplement: Supplementary file 2 — Supplementary Material 2 [file 12879_2026_13318_MOESM2_ESM.docx]

Supplement Table 1. Compared the baseline date of patients who retained and lost to follow-up.

|  | Retained  ( n = 157) | Lost to follow-up  ( n =135) | *P* value |
| --- | --- | --- | --- |
| Age, yr, mean ± SD | 59.1 ± 17.6 | 57.3 ± 14.3 | 0.16 |
| Male sex | 53 (33.8%) | 44 (32.6%) | 0.78 |
| BMI, kg/m^2^ | 22.7 ± 5.8 | 23.4 ± 3.2 | 0.08 |
| Smoking | 46 (29.3 %) | 36 (26.7%) | 0.56 |
| Alcohol misuse | 30 (19.1 %) | 37 (27.4%) | 0.12 |
| **Scores** | | | |
| APACHE II score | 21.9 ± 6.8 | 22.3 ± 4.9 | 0.57 |
| SOFA score | 9.35 ± 4.6 | 8.8 ± 4.2 | 0.29 |

Definition of abbreviations: BMI, Body Mass Index;APACHE II, Acute Physiology and Chronic Health Evaluation II; SOFA, Sequential Organ Failure Assessment Score.

Data are presented as the mean ± SD or number (percentage) of patients.

Supplementary Table 2. Sensitivity Analysis for the Association Between Sepsis and Osteoporosis Risk Under Worst-Case Scenarios of Loss to Follow-up.

| Analysis Scenario | Sepsis Group Osteoporosis, n/N (%) | Non-Sepsis Group Osteoporosis, n/N (%) | P-value | Odds Ratio (95% CI) |
| --- | --- | --- | --- | --- |
| **Primary Analysis (Observed Data)**  **Worst-Case Scenario 1** | 49/79 (62.0%) | 13/78 (16.7%) | <0.001 | 8.2 (3.9 – 16.9) |
| All lost sepsis patients: **No event** | 49/155 (31.6%) | 72/137 (52.6%) | 0.03 | 0.4 (0.3 – 0.7) |
| All lost non-sepsis patients: **Event** |  |  |  |  |
| **Worst-Case Scenario 2** |  |  |  |  |
| All lost sepsis patients: **Event** | 125/155 (80.6%) | 13/137 (9.4%) | <0.001 | +infinity (302.9 – +infinity) |
| All lost non-sepsis patients: No event |  |  |  |  |

Data are presented as the mean ± SD or number (percentage) of patients.

**Primary Analysis**: Data from the 157 patients who completed follow-up.

**Scenario 1**: Assumes the most unfavorable bias **against** the primary finding. All 76 lost sepsis patients did **not** develop osteoporosis, while all 59 lost non-sepsis patients **did** develop osteoporosis.

**Scenario 2**: Assumes the most unfavorable bias **in favor of** the primary finding. All lost sepsis patients **did** develop osteoporosis, while all lost non-sepsis patients did **not**.

**Interpretation of Sensitivity Analysis:** The two worst-case scenarios above delineate the potential boundaries of attrition bias. Scenario 1 (the assumption most unfavorable to the primary finding) resulted in a reversal of the association direction, indicating that our conclusion could be susceptible to extreme systematic bias in follow-up. However, the assumption underlying Scenario 1—that none of the lost sepsis patients but all of the lost non-sepsis patients developed osteoporosis—is highly implausible in practice. Considering the lack of significant differences in baseline characteristics between the retained and lost-to-follow-up groups (Supplementary Table 1), the loss to follow-up is more likely to be non-differential. Therefore, while the sensitivity analysis acknowledges that the observed association could theoretically be attenuated under extreme conditions, the strong association between sepsis and increased osteoporosis risk identified in the primary analysis remains informative under more realistic assumptions about the missing data.
