# Supplementary material for: Long-term bone metabolism outcomes in critically Ill patients with sepsis: a prospective series study
Source: BMC Infect Dis. 2026 Apr 17;26:1045. doi: 10.1186/s12879-026-13318-2 (PMC13217769; doi:10.1186/s12879-026-13318-2)
Supplement: Supplementary file 3 — Supplementary Material 3 [file 12879_2026_13318_MOESM3_ESM.docx]

STROBE Statement Compliance Report

Manuscript Title: Long-Term Bone Metabolism Outcomes in Critically Ill Patients with Sepsis: A Prospective Series Study

This report confirms that the aforementioned manuscript has been prepared in accordance with the Strengthening the Reporting of Observational Studies in Epidemiology (STROBE) Statement guidelines for cohort studies. Considerations from the STROBE-Nut extension for nutritional epidemiology have also been incorporated where applicable.

1. Title and Abstract

Item 1a: The study design is indicated in the title as "A Prospective Series Study".

Item 1b: The abstract provides a structured and informative summary under the headings Background, Methods, Results, and Conclusions, accurately reflecting the study's design and key findings.

2. Introduction

Item 2: The scientific background and rationale are thoroughly explained in the BACKGROUND section, covering long-term morbidity in sepsis survivors, the vulnerability of bone metabolism, the role of vitamin D, and existing knowledge gaps.

Item 3: Specific objectives and prespecified hypotheses are clearly stated in the final paragraph of the BACKGROUND section.

3. Methods

Item 4: Key elements of the study design (setting, timeframe, hybrid retrospective-prospective design) are presented early in the METHODS: Patients and study design section.

Item 5: The setting (First Affiliated Hospital of Xi'an Jiaotong University), relevant dates (recruitment: 2008-2018; follow-up ending: 2023), and periods of data collection are described in the METHODS: Patients and study design section.

Item 6a: Eligibility (inclusion/exclusion) criteria and participant selection are detailed in METHODS: Patients and study design. Participant flow is illustrated in Figure 1 and detailed in the RESULTS.

Item 6b: For the propensity score matched analyses, matching criteria (1:1 ratio, specific covariates) and the number of participants in matched groups are provided in the Statistical analysis section and RESULTS.

Item 7: All key outcomes (osteoporosis, mortality), exposures (sepsis, vitamin D status), and potential confounders are defined in the METHODS: Definition and Data collection sections, with reference to established diagnostic criteria (Sepsis-3, Endocrine Society, WHO).

Item 8: Sources of data (medical records, structured questionnaires) and detailed assessment methods for all variables, including laboratory assays and DXA scanning, are described in the METHODS: Data collection and Definition sections.

Item 9: Efforts to address bias include the use of propensity score matching (detailed in Statistical analysis) and a comparison of baseline characteristics between patients retained and lost to follow-up (mentioned in RESULTS, Supplementary Table 1). A sensitivity analysis for attrition bias has been added (Supplementary Table 2) in response to reviewer comments.

Item 10: The method for determining study size, based on an “a priori” power calculation, is explained in the Statistical analysis section.

Item 11:The handling of quantitative variables (presented as mean ± SD or median [IQR]) and the rationale for categorizing vitamin D (using a pre-specified cut-off of <20 ng/mL) are described in the “Statistical analysis” and “Definition” sections.

Item 12a: All statistical methods, including descriptive statistics, comparative tests, propensity score matching (with algorithm, caliper, and balance diagnostics using Standardized Mean Differences), and regression, are detailed in the Statistical analysis section. Covariate selection for matching was based on clinical relevance and causal reasoning.

Item 12b: The pre-specified subgroup analysis stratifying sepsis patients by vitamin D status is an integral part of the study design (Phase 3) and is reported in the RESULTS.

Items 12c & 12d: The handling of missing data and loss to follow-up is addressed in the METHODS: Patients and study design section (definitions and reasons). The analysis is based on available data, and the impact of loss to follow-up is examined via baseline comparison and a sensitivity analysis.

Item 12e: A sensitivity analysis to assess the robustness of findings to attrition bias has been conducted and is reported in Supplementary Table 2.

4. Results

Item 13a: Numbers of individuals at each stage of the study (assessed, excluded, included, followed, analyzed) are reported in the RESULTS sections and summarized in Figure 1.

Item 13b: Reasons for exclusion and non-participation are provided in the METHODS and RESULTS sections.

Item 14a: Characteristics of study participants are comprehensively presented in Tables 1, 2, and 3.

Item 14b: The number of participants with missing data for each variable is not explicitly tabulated. The manuscript states that patients with incomplete baseline laboratory data were excluded, which is acknowledged as a reporting limitation.

Item 15: The numbers of outcome events (osteoporosis) and summary measures of follow-up time (median, IQR) are reported throughout the RESULTS sections.

Item 16a: Both unadjusted comparisons (p-values) and confounder-adjusted estimates from matched analyses are provided. The key adjusted association (Odds Ratio, 95% Confidence Interval) between vitamin D deficiency and osteoporosis in sepsis patients is reported in Table 4. Confounders adjusted for via matching are listed in the Statistical analysis section.

Item 16b: The category boundary for the primary exposure (vitamin D deficiency: <20 ng/mL) is stated in the Definition section.

Item 17: Other analyses, including the pre-specified subgroup analysis (Table 3, Table 4) and sensitivity analysis (Supplementary Table 2), are reported.

5. Discussion

Item 18: Key results are summarized with reference to the study objectives in the first paragraph of the DISCUSSION.

Item 19: A dedicated paragraph on Limitations discusses four key potential sources of bias (attrition, temporality, follow-up duration, unmeasured confounders, single-center design) and their possible implications.

Item 20: A cautious overall interpretation of the results is provided, considering the study's objectives, limitations, and the context of existing literature (e.g., VITdAL-ICU trial, mortality debates). Mechanistic plausibility and clinical implications are discussed.

Item 21: The generalizability (external validity) of the findings is explicitly discussed within the Limitations paragraph.

6. Other Information

Item 22: The sources of funding are listed in the Funding section, and the role of the funder is indicated in the Authors' contributions statement.

STROBE-Nut Relevant Items

Item N1: Methods for assessing the nutritional exposure (25(OH)D) are described in detail in METHODS: Data collection and Definition. The supplementary Questionnaire.docx documents the assessment of supplement use and sun exposure during follow-up.

Item N2: The handling of supplementary data (vitamin D/calcium supplements) is acknowledged in the Limitations section, noting it was recorded via questionnaire but not included as a covariate in the primary model.

Item N3: The potential impact of measurement/classification error in nutritional status is discussed indirectly in the DISCUSSION, where heterogeneity in vitamin D deficiency definitions across studies is cited as a reason for discrepant findings.

Conclusion

This manuscript adheres to the reporting standards outlined in the STROBE Statement and the STROBE-Nut extension. All critical methodological details, results, and discussions of limitations are transparently presented within the main text, tables, figures, and supplementary materials.
